# Oxidative Stress Biomarkers as Preclinical Markers of Mild Cognitive Impairment: The Impact of Age and Sex

**DOI:** 10.3390/jpm15050171

**Published:** 2025-04-26

**Authors:** Stavroula Ioannidou, Magda Tsolaki, Argyrios Ginoudis, Androniki Tamvakis, Anthoula Tsolaki, Kali Makedou, Evgenia Lymperaki

**Affiliations:** 1 Department of Biomedical Sciences, International Hellenic University, 57400 Thessaloniki, Greece; sioanc@auth.gr; 2 Greek Association of Alzheimer’s Disease and Related Disorders (GAADRD), 54248 Thessaloniki, Greece; tsolakim@auth.gr (M.T.); tsolakianthoula@gmail.com (A.T.); 3 Laboratory of Neurodegenerative Diseases, Center for Interdisciplinary Research and Innovation (CIRI), Aristotle University of Thessaloniki, 54124 Thessaloniki, Greece; 4 School of Veterinary Medicine, Aristotle University of Thessaloniki, 54627 Thessaloniki, Greece; agkinou@vet.auth.gr; 5 Laboratory of Ecology and System Dynamics, Department of Marine Sciences, University of Aegean, 81100 Mytilene, Greece; atamvaki@mar.aegean.gr; 6 Laboratory of Biochemistry, School of Medicine, AHEPA University Hospital, Aristotle University of Thessaloniki, 54636 Thessaloniki, Greece

**Keywords:** oxidative stress, serum, cerebrospinal fluid, reactive oxygen species, malondialdehyde, amyloid, tau protein

## Abstract

**Background:** Reactive oxygen species (ROS) are involved in the pathophysiology of neurodegeneration and cognitive decline, indicating the potential use of oxidative stress biomarkers for early diagnosis. Mild cognitive impairment (MCI) is defined as a cognitive decline beyond normal aging, without significant impact on daily functioning, and is considered an important stage of early detection of neurodegeneration. This study aimed to investigate serum and cerebrospinal fluid (CSF) levels of oxidative stress biomarkers, total ROS and malondialdehyde (MDA) in patients with MCI to evaluate their utility in the early diagnosis of Alzheimer’s disease (AD). Levels of oxidative stress biomarkers were also assessed according to age and sex, as well as their correlation with the established CSF biomarkers, including amyloid-beta (Aβ40, Aβ42 and Aβ42/Aβ40 ratio), phosphorylated tau protein (p-tau) and total tau (t-tau). **Methods:** A total of 114 adults were divided into three groups: MCI (A−) patients with a normal CSF Aβ42/Aβ40 ratio (*n* = 38), MCI (A+) patients with an abnormal Aβ42/Aβ40 ratio (*n* = 38) and healthy cognitive function individuals with a normal Aβ42/Aβ40 ratio (*n* = 38). Established CSF biomarkers were conducted using an automated immunochemical method, while total ROS levels were measured by fluorometry and MDA levels were determined by competitive inhibition enzyme immunoassay. **Results:** A statistically significant difference was observed in CSF MDA levels (*p* < 0.05) and serum ROS levels (*p* < 0.05) between the study groups. Sex analysis revealed significantly higher levels of CSF MDA levels in the MCI (A+) males’ group (*p* < 0.05). In terms of age categorization, serum MDA levels were markedly higher in the MCI (A+) group of older patients (*p* < 0.01). **Conclusions:** These findings highlight the importance of individualized approaches, including investigation of oxidative stress biomarkers profile to prevent and manage individuals in the early stages of MCI, considering demographic factors.

## 1. Introduction

Mild cognitive impairment (MCI) is considered an intermediate stage between normal aging and dementia, particularly Alzheimer’s disease (AD). MCI is a clinical condition characterized by a noticeable decline in cognitive functions, such as memory and executive abilities, that is greater than expected for a person’s age and level of educational background, without significant impact on daily activities [1]. The identification of biomarkers that can predict which individuals with MCI will progress to AD is essential for early intervention and prevention strategies.

The clinical diagnosis of cognitive decline such as MCI, AD and other neurodegenerative diseases includes physical examination, cognitive assessments, brain imaging techniques and cerebrospinal fluid (CSF) biomarkers analyses [2,3]. The established CSF biomarkers are amyloid-beta (Aβ40, Aβ42 and Aβ42/Aβ40 ratio), phosphorylated tau (p-tau) and total tau protein (t-tau). Specifically, low levels of Aβ42 or Aβ42/Aβ40 ratio are known as indicators of cortical amyloid-beta deposition, and/or increased concentration of t-tau and/or p-tau are associated with the degree of neurodegeneration and consequently with the progression of the disease [4,5,6,7]. A recent longitudinal study has shown that CSF biomarkers begin to deviate from normal levels up to 18 years before clinical diagnosis of AD. In particular, CSF Aβ42 levels began to diverge from cognitively normal individuals approximately 18 years before clinical diagnosis, Aβ42/Aβ40 ratio at 14 years, p-tau at 11 years and t-tau at 10 years before diagnosis, highlighting their potential role in early diagnosis and progression of the disease [8].

Oxidative stress refers to the imbalance between the production and accumulation of reactive oxygen species (ROS) in a cell or tissue and the capacity of the organism to detoxify these detrimental products [9,10]. ROS are essential for maintaining homeostasis and regulating vital cellular functions at normal levels; however, they may lead to cellular and molecular damage at excessive levels. The reaction of ROS with lipids, in particular, polyunsaturated fatty acids, leads to lipid peroxidation, a process that produces aldehyde products capable of damaging proteins, compromising cell membranes and disrupting the integrity of DNA [11]. These molecular changes have extensive implications, particularly in the framework of neurodegenerative diseases [10]. In several neurodegenerative diseases, ROS overproduction is often associated with mitochondrial dysfunction. The accumulation of Aβ oligomers in mitochondrial membranes disrupts the electron transport chain, leading to excessive ROS production [12]. These contribute to DNA degradation, protein oxidation and lipid peroxidation, leading to a vicious cycle of oxidative damage and cellular dysfunction [12,13]. In particular, lipid peroxidation plays a pivotal role in the early stages of MCI and in the pathophysiology of AD, as it produces reactive aldehyde compounds that further damage neuronal structures and activate kinases involved in tau protein (tau) hyperphosphorylation [14]. These molecular cascades result in neuronal apoptosis and neuroinflammation, key factors in the progression of the disease [11].

New studies aim to investigate less invasive blood diagnostic biomarkers for the disease [15,16,17,18]. Research studies support and suggest the involvement of oxidative stress markers in the early diagnosis of the disease [19,20,21]. Specifically, individuals with MCI have higher levels of ROS and oxidative damage markers compared to cognitively healthy individuals. Research suggests that these elevated levels of ROS contribute to Aβ deposition and hyperphosphorylation of tau proteins, which are the main pathogenesis hypothesis of AD [22,23]. Notably, oxidative stress and its by-products, such as malondialdehyde (MDA), are observed in the early stages of cognitive decline, making them potential biomarkers for predicting the progression of MCI to AD [24]. This relationship highlights the critical need to identify and moderate oxidative stress as a novel strategy to predict or prevent the onset of neurodegenerative diseases [24,25]. It is essential to examine the influence of demographic factors background on oxidative stress and MCI.

Sex differences in oxidative stress and cognitive impairment have been extensively studied, with evidence suggesting that men generally have higher levels of oxidative damage than women [26,27,28,29]. This difference may be attributed to sex hormones, as estrogen has demonstrated neuroprotective and antioxidant effects. Postmenopausal women experience a decline in estrogen levels, which is associated with increased oxidative stress and a higher risk of developing AD [29]. Estrogen therapy, when prescribed alone, without progesterone, specifically for preventing Alzheimer’s disease and dementia, appears to be more beneficial for healthy, cognitively intact peri-menopausal women under the age of 65 years [27].

Additionally, more women are likely to be affected by AD due to longer life expectancy, although differences in incidence rates remain unclear and may vary by region and by type of dementia [30]. Furthermore, observational studies suggest a link between low testosterone levels and increased oxidative stress in men [30,31]. These highlight the complex interaction between hormonal alterations, oxidative stress and cognitive function, emphasizing the need for sex-specific studies in cognitive decline.

In addition to genetic background and sex, cognitive function can be influenced by various socio-cultural and economic factors. The effect of differences in educational attainment on cognitive decline increases with age, with women showing a steeper decline in cognitive function than men [32,33,34]. This difference highlights the need for further research to determine the socio-cultural and economic impact of cognitive decline. Higher cognitive capacity may help individuals compensate for neuronal loss by enabling more efficient cognitive networks, potentially delaying the onset of MCI and dementia [32]. Higher levels of education are often associated with healthier lifestyles, including better dietary habits and increased physical activity, which may reduce oxidative stress and support cognitive resilience [35].

Age is a critical factor in oxidative stress and the development of MCI. According to 2024 Alzheimer’s disease facts and figures, the percentage of people with Alzheimer’s dementia increases dramatically with age: 5% of individuals aged 65 to 74, 13.2% of those aged 75 to 84, and 33.4% of those aged 85 or older have Alzheimer’s dementia [36]. Aging is associated with a progressive decline in the efficiency of antioxidant defenses, leading to increased susceptibility to oxidative damage [37,38]. The accumulation of oxidative stress over the years contributes to neuronal dysfunction, synaptic loss and the deposition of pathological proteins that occur in MCI and AD [37,39]. In view of the increasing prevalence of cognitive disorders in the aging population, an understanding of the relationship between oxidative stress and age-related cognitive decline is crucial for the potential preventive interventions.

MCI is considered the earliest clinical stage of dementia, particularly in AD. Reliable and accessible biomarkers for the early identification of MCI remain limited. Oxidative stress has been increasingly implicated in the pathogenesis of AD, but its role in the early stages of cognitive decline remains unclear. This study focuses on the evaluation of oxidative stress biomarkers, MDA and ROS, in serum and CSF of individuals with MCI, to assess their potential use in early diagnosis of AD. In addition, the influence of demographic factors such as age and sex on oxidative stress levels remains underexplored in MCI patients and may be essential for the development of personalized diagnostic and therapeutic strategies. Therefore, this study aims to investigate a combined biomarker approach, including both serum and CSF levels of oxidative stress markers, considering demographic factors, in order to predict the progression of MCI to AD.

## 2. Materials and Methods

### 2.1. Study Population

In this study, 114 adults, 60 women and 54 men, aged 55–90 years old were included. All participants were from Northern Greece and were examined by a neurologist from the Greek Association of Alzheimer’s Disease and Related Disorders. Participants in this study, including healthy controls, were selected based on specific inclusion criteria determined by screening assessments, medical history, laboratory tests and physical examinations. The healthy controls volunteered for the study and, although some had a family history of dementia, none of them had symptoms. They participated as part of a preventive screening check. All participants were assessed by a neurologist using screening tools, including (a) the Mini-Mental State Examination, which measures global cognitive function [40], (b) the Functional Rating Scale for Dementia Symptoms, which assesses functionality [41] and (c) the Hamilton Depression Rating Scale [42]. Personal details, including age, sex, body mass index (BMI) and family history, as well as findings from physical and neurological examinations, were documented by the study neurologist and stored in the database of the Greek Association of Alzheimer’s Disease and Related Disorders.

Participants were initially divided into two groups: 76 individuals with MCI and 38 healthy controls, based on the screening assessments. These participants were further divided into three groups based on their cerebrospinal fluid amyloid status: 38 MCI patients (A−) with a normal CSF Aβ42/Aβ40 ratio, 38 MCI patients (A+) with an abnormal CSF Aβ42/Aβ40 ratio and 38 healthy controls (A−) with a normal CSF Aβ42/Aβ40 ratio. The cut-off criteria for positivity were an Aβ42/Aβ40 ratio of less than 0.058 and at least two other abnormal biomarkers: Aβ42 < 638 pg/mL, Aβ40 > 11,017 pg/mL, t-tau > 404 pg/mL and p-tau > 52.1 pg/mL [43]. Individuals with positive CSF biomarkers were classified as MCI (A+) and were considered to be at higher risk of developing AD later in life. None of the participants were taking any medications, supplements or other drugs at the time of the study. Participants had already been analyzed and categorized according to these criteria and were recruited by random selection from the referenced sample bank.

The characteristics of the study groups are presented in terms of sex, age, BMI and family history, in the following table (Table 1). Sex was categorized into male and female groups. Age was divided into two categories: under 75 years and 75 years or older. This age cut-off was chosen because the incidence and prevalence of AD increase significantly with age. Approximately 80% of people aged 75 years and older have AD. The incidence of AD rises dramatically with age, from 2 per 1000 people aged 65–74 to 37 per 1000 people aged 85 and over [44].

### 2.2. Sample Collection

Samples were obtained and provided by the Greek Association of Alzheimer’s Disease and Related Disorders. Following patients’ screening assessments, which included medical history, laboratory tests and physical examination, the neurologist recommended the selection of CSF and blood samples. CSF and blood samples were collected in the morning hours under fasting conditions. All samples were sourced from the Association’s sample bank and stored at −80 °C. Hemolyzed samples were excluded from the study. Prior to the experiment, all samples, blood serum and CSF, were incubated at room temperature.

A stratified random sampling was followed using data and samples from the biobank of the Greek Association of Alzheimer’s Disease and Related Disorders, which contains a large number of samples. Based on a statistical analysis, a sample size of more than 30 participants per group was considered sufficient for the primary analyses. This approach ensured balanced group sizes, improved comparability and increased statistical power. A total of 114 participants were randomly selected, comprising three equal groups: 38 MCI patients with a normal Aβ42/Aβ40 ratio, 38 MCI patients with an abnormal Aβ42/Aβ40 ratio and 38 healthy individuals with a normal Aβ42/Aβ40 ratio. 

### 2.3. Cerebrospinal Fluid Established Biomarkers Analyses

CSF levels of Aβ40, Aβ42, Aβ42/Aβ40 ratio, t-tau and p-tau were measured using an automated immunochemical method that was performed with the Lumipulse G1200 automated analyzer (Fujirebio Inc., Tokyo, Japan).

### 2.4. Reactive Oxygen Species Analysis

The determination of ROS was performed by the fluorimetric method (Abcam, Cambridge, United Kingdom), using the Infinite 200 PRO fluorimeter (TECAN Trading AG, Mannedorf, Switzerland). In this method, H2DCFDA fluorogenic dye was used as a reagent, which reacted with the hydroxyl, peroxyl and other reactive oxygen species; this dye was oxidized, leading to the production of the fluorescent compound 2′,7′-dichlorodihydrofluoroscein (DCF). DCF was highly fluorescent and was detected by fluorescence spectroscopy with excitation/emission at 485 nm/535 nm. Ninety-six black-walled microtitration plates were used in the fluorimeter. The fluorescence intensity was proportional to the ROS levels in the sample.

### 2.5. Malondialdehyde Analysis

MDA levels were determined using a competitive inhibition enzyme immunoassay with an enzyme-linked immunosorbent assay (ELISA) kit from ELK Biotechnology Co., Ltd. (Denver, CO, USA). A commercially available 96-well microtiter plate had been pre-coated with the Human MDA protein. Standards and samples were added to appropriate microtiter plate wells, followed by the addition of biotin-conjugated specific to Human MDA. After a one-hour incubation, avidin conjugated to horse-radish peroxidase was added. Following another one-hour incubation, TMB substrate solution was added. The enzyme–substrate reaction was terminated using sulfuric acid solution and the color change was measured spectrophotometrically at a wavelength of 450 nm. MDA concentrations in the sample were calculated based on a standard curve.

### 2.6. Statistical Analyses

A statistical software package R Studio (Posit Software 2024, R Studio Desktop for Windows, Version 2024.04.1+748, Boston, MA, USA) was used for the statistical analyses. Descriptive statistics were conducted for each sociodemographic variable. The sample data were checked for normality with the Kolmogorov–Smirnov test and appropriate statistical tests were chosen for analyses. In all of the statistical analyses, the level of significance was set at *p* < 0.05.

### 2.7. Ethical Considerations

The study adhered to Good Clinical Practice guidelines, the Declaration of Helsinki. Ethical approval of the study was obtained from the bioethics committee of the Greek Society for Alzheimer’s Disease and Related Disorders 175/2024, 4 July 2024. All participants signed an informed consent about using their samples in research. The confidentiality was strictly maintained and participants’ personal privacy was fully respected.

## 3. Results

### 3.1. Median Values of CSF Established Biomarkers and Oxidative Stress Biomarkers Within the Study Groups

**Figure 1:**
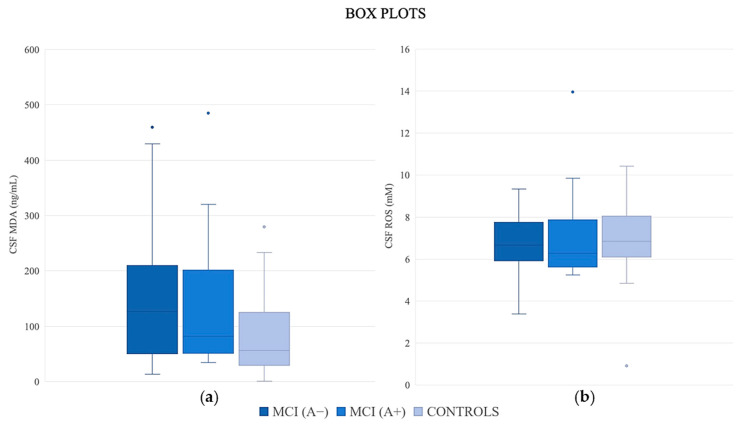
Box plots for oxidative stress biomarkers (**a**) CSF ROS and (**b**) CSF MDA in the study groups. The bottom and top of the box represent the 25th and 75th percentile. Dashed lines encompass the range. Dots represent outlier data points.

**Figure 2:**
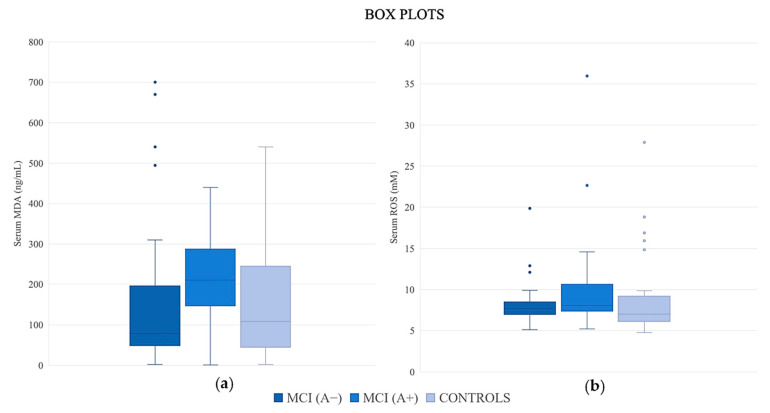
Box plots for oxidative stress biomarkers (**a**) serum ROS and (**b**) serum MDA in the study groups. The bottom and top of the box represent the 25th and 75th percentile. Dashed lines encompass the range. Dots represent outlier data points.

Table 2, Figure 1 and Figure 2 present the median values of CSF-established biomarkers and oxidative biomarkers (MDA and ROS) in the three groups: MCI (A−), MCI (A+) and controls (A−). As expected, statistically significant differences were observed in the levels of Aβ42 (*p* < 0.001), Aβ42/Aβ40 (*p* < 0.001), p-tau (*p* < 0.001) and t-tau (*p* < 0.001).

The median values of CSF MDA revealed significant differences among the study groups [MCI (A−): 126.50 ng/mL vs. MCI (A+): 82.00 ng/mL vs. controls (A−): 56.00 ng/mL, *p* < 0.05)]. CSF MDA levels were elevated in MCI (A−) patients compared to both MCI (A+) and cognitively healthy controls. Serum ROS levels also had a significant difference among the study groups [MCI (A−): 7.71 mM vs. MCI (A+): 8.07 mM vs. controls (A−): 7.02 mM, *p* < 0.05]. Serum ROS levels were elevated in MCI (A−) patients and MCI (A+) compared to healthy controls. The median values of CSF ROS and serum MDA did not reveal significant differences.

### 3.2. Correlations of CSF Established Biomarkers and Oxidative Stress Biomarkers

Table 3 presents the correlation between CSF-established biomarkers and oxidative stress biomarkers, using the Spearman correlation test. There was not observed any statistically significant correlation between the biomarkers. All oxidative stress biomarkers revealed a non-statistically significant negative correlation with the Aβ42/Aβ40 ratio.

### 3.3. Median Values of Oxidative Stress Biomarkers, According to Sex

The groups were divided by sex, men and women. Table 4 presents the median values of CSF and serum oxidative biomarkers (MDA and ROS) in the three groups: MCI (A−), MCI (A+) and controls (A−). Wilcoxon test and *t*-test were performed to test whether the outcomes of the groups differed from each other significantly. CSF MDA levels in MCI (A+) patients differed significantly among the groups (*p* < 0.05). In males with MCI (A+), the CSF MDA levels were markedly higher (157.00 ng/mL) than in female MCI (A+) patients (65 ng/mL). As well as males with MCI (A−) had higher levels of CSF MDA (133.00 ng/mL) than female MCI (A−) patients (80.50 ng/mL). In the control group, higher levels of CSF MDA was observed in females (68.00 ng/mL) than in males (56.50 ng/mL). Serum MDA showed a similar trend, it was observed higher levels of serum MDA in males with MCI (A+) and MCI (A−) patients (236.50 ng/mL and 136.00 ng/mL) than in female MCI (A+) and MCI (A−) patients (162.50 ng/mL and 62.50 ng/mL, respectively). In the control group, higher levels of serum MDA was observed in females (134.00 ng/mL) than in males (53.50 ng/mL).

### 3.4. Median Values of Oxidative Stress Biomarkers, According to Age

The groups were divided by age, <75 years old and ≥75 years old. Table 5 presents the median values of CSF and serum oxidative biomarkers (MDA and ROS) in the three groups: MCI (A−), MCI (A+) and controls (A−). Kruskal–Wallis test and one-way ANOVA were performed to test whether the outcomes of the groups differed from each other significantly. CSF MDA levels are elevated in younger people (<75 years old) with MCI (A+) (100.00 ng/mL) and in MCI (A−) patients (149.00 ng/mL), but not in healthy controls (55.25 ng/mL). Serum MDA levels differed significantly among the age categories in MCI (A+) patients (165 ng/mL vs. 240 ng/mL, *p* < 0.001). Serum MDA levels are elevated in older people with MCI (A+) and healthy controls, but not in MCI (A−) patients (240 ng/mL and 193.00 ng/mL vs. 66.85 ng/mL). No other significant differences were observed.

## 4. Discussion

This study investigated the relationship between oxidative stress biomarkers and established CSF biomarkers in patients with MCI, indicating the potential use of oxidative stress biomarkers for early diagnosis. The findings of this study highlight the significant role of oxidative stress in neurodegeneration, supporting the hypothesis that oxidative damage is a key contributor to cognitive decline. Additionally, the results of this study demonstrate that sex and age significantly influence oxidative stress levels, suggesting the need for personalized approaches in diagnosing and managing cognitive impairment.

The results of this study show that patients with MCI exhibit significant alterations in oxidative stress biomarkers. In particular, CSF MDA levels were elevated in MCI (A−) patients compared to both MCI (A+) and cognitively healthy controls, while serum ROS levels were elevated in MCI (A+) patients and (A−) compared to healthy controls. This suggests that CSF MDA and serum ROS biomarkers may be an early marker of cognitive impairment, preceding amyloid pathology. The fact that MCI (A−) patients—who do not yet exhibit abnormal amyloid-beta (Aβ) deposition—already show increased oxidative stress status indicates that lipid peroxidation and oxidative damage may play a critical role in the earliest stages of neurodegeneration, even before detectable amyloid accumulation. This finding aligns with previous studies that propose oxidative stress as an initiator of neuronal damage rather than merely a consequence of amyloid pathology [45,46].

Given that CSF MDA, a marker of lipid peroxidation, is significantly elevated in MCI (A−) patients, it may serve as a potentially earlier biomarker for identifying individuals at risk of developing MCI and eventually progressing to AD. Similarly, increased serum ROS levels further support the systemic nature of oxidative stress in cognitive decline. These findings emphasize the need for further longitudinal studies to determine whether oxidative stress markers could be used in clinical settings for early detection and intervention, potentially enabling more effective preventive strategies for neurodegenerative diseases.

Elevated oxidative stress levels have been consistently observed in neurodegenerative conditions and are thought to contribute to disease progression by promoting Aβ accumulation and tau hyperphosphorylation [45,46]. Studies have shown increased brain oxidative stress and lipid peroxidation markers such as MDA in MCI patients, linking oxidative stress to AD progression [47,48]. This study supports these findings by demonstrating significant differences in MDA between MCI patients and healthy controls, suggesting its potential as an AD risk biomarker. However, the lack of significant differences in serum MDA, in contrast to CSF, may be due to the selective permeability of the blood–brain barrier (BBB), peripheral systemic factors and inherent biomarker dynamics between central and peripheral compartments. These factors highlight the importance of considering compartment-specific biomarkers when studying oxidative stress and amyloid pathology in neurodegenerative diseases [49,50].

The findings of this study reveal notable sex differences in oxidative stress levels, particularly in MCI patients. Males exhibited significantly higher CSF MDA levels than females, likely due to distinct pathophysiology mechanisms of the disease in men and women, possibly linked to lifestyle, genetic predisposition and/or socio-cultural factors. This discrepancy may reflect different biological pathways contribute to disease progression. CSF and serum MDA levels followed a similar trend, with higher levels in males with MCI than in females with MCI, whereas in the healthy control group, females had higher CSF and serum MDA levels than males. This aligns with prior research suggesting that men are more susceptible to oxidative damage due to lower endogenous antioxidant capacity [26]. Sex hormones, particularly estrogens, have been shown to exert neuroprotective effects by modulating mitochondrial function and reducing oxidative stress [51]. The decline in estrogen levels after menopause may partially explain why women have a higher lifetime risk of developing AD, despite exhibiting lower oxidative stress levels in earlier stages [52].

Conversely, testosterone has been implicated in the regulation of oxidative stress in men. Lower testosterone levels in aging males have been associated with increased oxidative stress, inflammation and cognitive decline [30]. Testosterone has been shown to have neuroprotective and antioxidant roles, particularly under certain circumstances [53]. Several studies have reported that testosterone may exhibit pro-oxidant activity, in high oxidative stress environments, by increasing neuroinflammation and oxidative damage through pathways such as nuclear factor kB (NF-kB) and cyclooxygenase-2 (COX-2) activation [54]. The testosterone’s immunosuppressive effects may be mediated through increased oxidative stress. Testosterone therapy has been shown to reduce systemic oxidative stress in older men, further supporting the hypothesis that hormonal balance plays a crucial role in neuroprotection [55]. These findings highlight the need for sex-specific therapeutic approaches in addressing oxidative stress-related neurodegeneration.

In addition, previous studies have suggested that sex differences in dementia prevalence may be influenced by both biological and social factors [33]. Women tend to live longer, which increases their risk of developing Alzheimer’s disease. Men may experience more severe oxidative stress-related damage earlier in life [34]. This suggests that different pathological mechanisms may drive cognitive decline in men and women, reinforcing the need for tailored interventions. Furthermore, this study aligns with previous research suggesting that systemic oxidative stress is closely linked to aging [56]. Oxidative damage accumulates with age due to mitochondrial dysfunction, reduced antioxidant defenses and increased inflammatory responses, all of which contribute to neuronal vulnerability [57]. Given the association between oxidative stress and cognitive decline, future studies should explore whether antioxidant-based interventions could mitigate neuronal damage and delay disease progression.

Aging is a well-established risk factor for neurodegeneration, with oxidative stress playing a central role in age-related cognitive decline [45]. This study found that serum MDA levels were significantly higher in older MCI (A+) patients (≥75 years old) compared to younger MCI (A+) patients. Serum MDA levels were also elevated in older people with MCI (A+) and healthy controls, but not in MCI (A−) patients. These findings are consistent with previous research indicating that aging leads to increased lipid peroxidation and oxidative DNA damage, which contribute to neuronal dysfunction [58]. In contrast, this study found non statistically significant higher CSF MDA levels in younger people (<75 years old) with MCI (A+) and MCI (A−), but not in healthy controls, considering age as an additional prognostic factor.

It is widely recognized that mitochondrial dysfunction is one of the key mechanisms that contribute to aging and functional decline [45]. As individuals age, mitochondrial efficiency declines, leading to reduced adenosine triphosphate (ATP) and excessive ROS production, reducing antioxidant capacity [45,56]. This oxidative imbalance can trigger neuroinflammation, synaptic dysfunction and apoptosis, all of which contribute to cognitive impairment [59]. Furthermore, oxidative stress is known to exacerbate vascular dysfunction, which is a significant contributor to both AD and vascular dementia [60].

The findings of this study align with studies suggesting that oxidative stress markers could serve as early predictors of cognitive decline. Elevated levels of MDA and 8-hydroxy-2′-deoxyguanosine (8-OHdG) have been linked to an increased risk of developing dementia in elderly populations [61]. Given the observed increase in oxidative stress with age, early interventions aiming at reducing oxidative damage—such as antioxidant supplementation, lifestyle modifications and pharmacological approaches—may help mitigate the risk of cognitive decline.

The significant differences in oxidative stress biomarkers between MCI patients and healthy controls suggest that oxidative stress plays a crucial role in neurodegeneration. The findings of this study support the use of oxidative stress markers, particularly CSF MDA and serum ROS, as potential adjunct biomarkers for early detection of cognitive impairment. Given the limitations of current diagnostic methods, integrating oxidative stress biomarkers with established CSF markers could enhance diagnostic accuracy and improve risk assessment for AD progression [46].

In addition, this study highlights the importance of considering sex- and age-related differences in oxidative stress for early diagnosis. The results of this study suggest that oxidative stress biomarkers could complement current diagnostic protocols for AD, particularly in individuals with MCI. These biomarkers, when combined with existing cognitive assessments and neuroimaging techniques, may help to identify individuals at risk earlier, providing a non-invasive and cost-effective tool for early diagnosis. Personalized approaches that take into account hormonal influences and age-related oxidative damage may improve the accuracy of biomarker-based detection. In addition, oxidative stress markers could help monitor disease progression and assess the effectiveness of therapeutic interventions, supporting more personalized treatment strategies Future research should investigate how these factors influence oxidative stress markers to improve early identification of individuals at risk for Alzheimer’s disease. It could also focus on investigating targeted interventions such as antioxidant therapies and hormone replacement strategies to mitigate oxidative stress-related neurodegeneration.

Despite its strengths, this study has several limitations that should be acknowledged. The cross-sectional design prevents from establishment of causal relationships between oxidative stress and cognitive decline. Longitudinal studies are needed to determine whether oxidative stress biomarkers can reliably predict MCI progression to AD. Additionally, the sample size of this study may limit the generalizability of the findings, particularly regarding sex- and age-related differences in oxidative stress biomarkers. Although the sample size was sufficient for the primary analyses, categorization by sex and age resulted in smaller subgroup sizes, potentially reducing the statistical power and limiting the ability to detect more subgroup-specific effects. This limitation may affect the robustness and generalizability of findings, particularly in the interpretation of sex- and age-related differences in oxidative stress biomarkers. Even though appropriate statistical methods were used to account for these limitations, the potential for reduced sensitivity in subgroup analyses remains. Future studies with larger, more diverse cohorts and stratified sampling designs will be essential to validate and extend the findings of this study, as well as to explore potential interactions between sex, age and oxidative stress biomarkers more thoroughly. Finally, while this study focused on MDA and ROS, future studies should include additional oxidative markers, to provide a more comprehensive understanding of oxidative damage in neurodegeneration.

## 5. Conclusions

In summary, the findings of this study highlight the critical role of oxidative stress in MCI and its potential implications for AD progression. The observed associations between oxidative stress biomarkers and established CSF markers suggest that oxidative damage is linked to amyloid pathology and neurodegeneration. These findings provide further evidence that oxidative stress is not just a consequence of Alzheimer’s pathology, but may actively contribute to its early development.

A particularly promising finding is the significant elevation of CSF MDA and serum ROS levels in MCI (A−) patients, even in the absence of amyloid pathology. This suggests that CSF MDA and serum ROS levels could serve as a potential prognostic biomarker for identifying individuals at an earlier stage of cognitive impairment, before the onset of amyloid accumulation. If validated in longitudinal studies, CSF MDA and serum ROS levels could enhance early identification of MCI individuals at risk for AD.

Additionally, the results of this study reveal sex and age-related differences in oxidative stress levels. These findings emphasize the need for a personalized approach to cognitive health, recognizing that biological sex and age may have an influence on the onset and progression of oxidative stress-related cognitive changes. These differences suggest that interventions related to oxidative stress may need to be tailored to individual demographic profiles in order to be more effective.

Longitudinal studies are needed to confirm the prognostic value of oxidative stress biomarkers and to establish their reliability in predicting progression from MCI to AD. Integration of these biomarkers into routine clinical assessment alongside established CSF and imaging markers may improve early detection and risk prediction. In addition, interventional trials focusing on the reduction of oxidative stress, through pharmacological agents, dietary strategies or lifestyle modifications, should investigate their potential role to delay or prevent cognitive decline, potentially offering new avenues for early intervention and neuroprotective treatments.

By advancing the understanding of oxidative stress in cognitive impairment and highlighting the potential use of CSF MDA and serum ROS as early biomarkers, this study paves the way for novel diagnostic and therapeutic strategies aiming at reducing oxidative damage and preserving cognitive function in aging populations. Future research should focus on longitudinal studies to determine whether oxidative stress biomarkers could be used as early biomarkers in MCI (A−) patients potentially even earlier than 18 years before clinical diagnosis, even before detectable changes in established CSF biomarkers.

## Figures and Tables

**Table 1:** Characteristics of the study groups.

	MCI (A−) *n* = 38	MCI (A+) *n* = 38	Controls *n* = 38
Sex (%)			
Men	50.00	47.37	44.74
Women	50.00	52.63	55.26
Age, mean ± SD (years)	73.00 ± 6.00	75.00 ± 5.00	61.00 ± 8.00
BMI (kg/m^2^)	27.6	26.2	27.7
Family history (%)			
Yes	65.79	47.37	44.74
No	34.21	52.63	55.26

Abbreviations: SD: standard deviation, BMI: body mass index.

**Table 2:** Median of CSF established biomarkers and oxidative stress biomarkers within the study groups.

Median Values				
Cerebrospinal Fluid Biomarkers	MCI (A−) *n* = 38	MCI (A+) *n* = 38	Controls (A−) *n* = 38	*p*-Values
Aβ42 (pg/mL)	900.00	581.00	1211.00	<0.001
Aβ40 (pg/mL)	10,865.50	12,387.50	11,994.00	0.156
Aβ42/Aβ40 ratio	0.09	0.04	0.11	<0.001
p-tau (pg/mL)	35.65	104.15	32.10	<0.001
t-tau (pg/mL)	297.00	639.00	249.00	<0.001
MDA (ng/mL)	126.50	82.00	56.00	<0.050
ROS (mM)	6.66	6.26	6.84	0.700
**Serum Biomarkers**				
MDA (ng/mL)	79.00	211.00	108.00	0.103
ROS (mM)	7.71	8.07	7.02	<0.050

Abbreviations: Aβ: amyloid-beta, p-tau: phosphorylated tau protein, t-tau: total tau protein, MDA: malondialdehyde, ROS: reactive oxygen species and CSF: cerebrospinal fluid.

**Table 3:** Spearman correlation of CSF established biomarkers and oxidative stress biomarkers.

Cerebrospinal Fluid Biomarkers	Aβ42	Aβ40	Aβ42/Aβ40 Ratio	p-tau	t-tau
MDA (ng/mL)	−0.102	−0.151	−0.051	−0.007	0.032
ROS (mM)	0.086	0.173	−0.018	0.164	0.122
**Serum Biomarkers**					
MDA (ng/mL)	−0.214	−0.071	−0.221	0.063	0.073
ROS (mM)	−0.095	0.139	−0.170	0.129	0.128

Abbreviations: Aβ: amyloid-beta, p-tau: phosphorylated tau protein, t-tau: total tau protein, MDA: malondialdehyde, ROS: reactive oxygen species and CSF: cerebrospinal fluid.

**Table 4:** Median values of oxidative stress biomarkers, according to sex.

Median Values						
	MCI (A−) *n* = 38		MCI (A+) *n* = 38		Controls (A−) *n* = 38	
Oxidative stress Biomarkers	Males *n* = 19	Females *n* = 19	Males *n* = 18	Females *n* = 20	Males *n* = 17	Females *n* = 21
**CSF**						
MDA (ng/mL)	133.00	80.50	157.00 *	65.00 *	56.50	68.00
ROS (mM)	6.42	6.84	6.00	7.02	7.02	6.74
**Serum**						
MDA (ng/mL)	136.00	62.50	236.50	162.50	53.50	134.00
ROS (mM)	8.10	7.45	8.07	8.05	7.18	6.78

Abbreviations: MDA: malondialdehyde, ROS: reactive oxygen species and CSF: cerebrospinal fluid. Note: * *p*-Value < 0.05.

**Table 5:** Median values of oxidative stress biomarkers in the study groups, according to age.

Median Values						
	MCI (A−) *n* = 38		MCI (A+) *n* = 38		Controls (A−) *n* = 38	
Oxidative Stress Biomarkers	Aged < 75 *n* = 19	Aged ≥ 75 *n* = 19	Aged < 75 *n* = 18	Aged ≥ 75 *n* = 20	Aged < 75 *n* = 21	Aged ≥ 75 *n* = 17
**CSF**						
MDA (ng/mL)	149.00	81.50	100.00	65.50	55.25	97.00
ROS (mM)	6.76	6.54	6.20	6.97	6.82	7.31
**Serum**						
MDA (ng/mL)	99.50	66.85	165.00 *	240.00 *	102.50	193.00
ROS (mM)	7.45	8.13	8.58	8.01	6.99	7.29

Abbreviations: MDA: malondialdehyde, ROS: reactive oxygen species and CSF: cerebrospinal fluid. Note: * *p*-Value < 0.01.

## Data Availability

The original contributions presented in this study are included in the article. Further inquiries can be directed to the corresponding author.

## Abbreviations

The following abbreviations are used in this manuscript:

Aβ	Amyloid-beta
AD	Alzheimer’s disease
ATP	Adenosine triphosphate
BBB	Blood–brain barrier
BMI	Body mass index
CSF	Cerebrospinal fluid
COX-2	Cyclooxygenase-2
DCF	2′,7′-dichlorodihydrofluoroscein
ELISA	Enzyme-linked immunosorbent assay
GAADRD	Greek Association of Alzheimer’s Disease and Related Disorders
MCI	Mild Cognitive impairment
MDA	Malondialdehyde
NF-kB	Nuclear factor kB
8-OHdG	8-hydroxy-2′-deoxyguanosine
p-tau	Phosphorylated tau protein
ROS	Reactive oxygen species
SD	Standard deviation
tau	Tau protein
t-tau	Total tau protein
